# Seeds or Parasites? Clinical and Histopathological Features of Seeds and Parasites in the Appendix

**DOI:** 10.5146/tjpath.2022.01586

**Published:** 2023-01-15

**Authors:** Gizem Issın, Fatih Demır, Hasan Aktug Simsek, Diren Vuslat Cagatay, Mahir Tayfur, Ali Kandemır, Mecdi Gürhan Balcı

**Affiliations:** Department of Pathology, Erzincan Binali Yildirim University, Mengucek Gazi Training and Research Hospital, Erzincan, Turkey; Eskisehir City Hospital, Eskişehir, Turkey; Department of Biology, Erzincan Binali Yildirim University, Faculty of Science and Art, Erzincan, Turkey

**Keywords:** Plant seeds, Enterobius vermicularis, Taenia subspecies, Foreign bodies, Appendicitis, Parasites

## Abstract

***Objective:*** Parasites and plant seeds may both be found in appendectomy specimens. Each plant seed has a different appearance and can thus exhibit wide variations under the microscope. Fragmented seeds may histologically mimic parasites. The differential diagnosis between seeds and parasites can be challenging in such cases. This study aimed to determine the incidence of parasites, seeds, and foreign bodies in appendectomy materials and highlight the most characteristic histopathological features associated with these structures.

***Material and Method:*** In this study, pathology slides of 9,480 patients, who underwent appendectomy between 2010 and 2021, were reviewed, and cases that contained parasites, seeds, or foreign bodies were identified. We reviewed the literature on seeds and parasites in appendectomy specimens.

***Results:*** Parasites were observed in 56 (0.6%) cases. Of these cases, 45 had *Enterobius vermicularis* (80%), and 11 had *Taenia* subspecies (20%). Plant seeds were observed in 47 cases (0.5%), and were macroscopically recognizable in 5 of them as olive, lemon, and cherry seeds. Parasites and seeds were usually observed in the lumen of appendix vermiformis, filled with abundant fecal materials.

***Conclusion:*** Seeds are seen in approximately 0.5% of the appendectomy specimens. Though rarely seen, the fragmented seed appearance may cause diagnostic difficulties. In this context, the key morphological features of parasites and plant seeds outlined in this study may be helpful in their differential diagnosis.

## INTRODUCTION

Parasitic infections of the gastrointestinal tract are common; nevertheless, parasites are rarely detected in appendectomy specimens (1). The most common parasitic agent in the appendix is *Enterobius vermicularis* (EV). Other parasites, such as *Taenia *subspecies (Taenia spp.), *Balantidium coli, Entamoeba histolytica, Schistosoma*, and *Ascaris lumbricoides*, are less common (2,3). If parasites are detected in appendectomy materials, stand-alone application of appendectomy is not sufficient for treatment. It may be necessary to run additional diagnostic tests and plan treatments.

Foreign bodies, undigested food residues, and plant seeds may occasionally be seen in appendectomy specimens (4–6). Each plant seed and food particle has a different appearance and can thus exhibit wide histological variations under the microscope. Given their rarity, non-expert pathologists may be unfamiliar with the histopathological features of such substances. Consequentially, the undigested food particles and seeds might be misdiagnosed as parasites, which leads to unnecessary treatment decisions.

In this study, we aimed to determine the incidence of parasites, plant seeds, and foreign bodies in appendectomy materials and highlight the most characteristic histopathological features of the most commonly identified seeds and parasites to provide sample reference images, which would be helpful in routine diagnosis.

## MATERIAL and METHOD

The pathology departments’ electronic records were reviewed to identify the patients that underwent appendectomy between January 2010 and January 2021. The hematoxylin and eosin (H&E)-stained slides of all cases were re-examined. Cases in which parasites, foreign bodies, and seeds were found were included in this study. The demographic data and histopathological features of the cases were documented.

The undigested materials in the appendectomy specimens were evaluated together with the botanist (A.K.). Cases with intact plant structures such as seed coat, endosperm, and embryo sections were categorized as the cases containing seeds. However, cases containing only fragmented or semi-digested plant structures were categorized as the cases containing food residues. The types of the seeds observed in these cases were identified by comparing them with reference images of seeds and plants in the catalogues (5–7).

We reviewed the literature to identify studies about parasites observed in appendectomy specimens. Methods, flow chart of literature review, inclusion and exclusion criteria were given in Appendix 1. The relevant studies are compiled in Table 1 (8–50). Also, we reviewed the literature to identify studies about seed observed in appendectomy specimens without any date or language restriction and compiled the relevant studies in Table 2

**Table 1:** Literature review summary table: Parasites in the appendix

**No**	**Author**	**Year**	**Country-City**	**Infected Case Number/Total Case Number**	**Parasite’s type**	**Female/Male**	**A. A/ Infected Cases**	**Age means or range (years)**
1	Egilmez et al. (8)	2000	Turkey, Sivas	83/847 (9.80%)	EV (25), Tenia spp. (38), TT (5), AL (8), Other (7)	42/46 [sic]	31/83 (37%)	21.5
2	Dorfman et al. (9)*	2003	Venezuela	62/830 (7.47%)	EV (7), TT (46), B. Coli (2), E. Histolytica (3), Other (4)	NS	41/62 (66%)	7-12
3	Arca et al. (10)*	2004	United States	21/1549 (1.36%)	EV (21)	11/10	15/21 (71%)	8.2
4	Yildirim et al. (11)	2005	Turkey, Adana	5/104 (4.81%)	EV (4), E. Histolytica (1)	NS	2/5 (40%)	42 ± 12.5
5	Fallah et al. (12)*	2006	Iran	38/5981 (1.38%) [sic]	EV (38)	25/13	NS	NS
6	Sah and Bhadani (13)	2006	Nepal	9/624 (1.44%)	EV (9)	6/3	3/9 (33%)	15
7	Aydin (14)	2007	Turkey, Antalya	6/190 (3.16%)	EV (4), Tenia spp. (2)	3/3	2/6 (33%)	18
8	Da Silva et al. (15)	2007	Brazil	24/1600 (1.5%)	EV (23), Tenia spp. (1)	9/15	12/24 (50%)	NS
9	Ramezani and Dehghani (16)	2007	Iran	144/5048 (2.85%)	EV (144)	NS	NS	20.4 ± 11.7
10	Chamisa (17)	2009	South Africa	25/324 (7.72%)	EV (3) AL (3) Schistosoma Spp. (12), TT (5), E. Histolytica (2)	NS	NS	NS
11	Al-Shadood et al. (18)	2009	Iraq	81/500 (16.2%)	EV (50) AL (3), G. Lamblia (10), TT (1), E. Histolytica (14), Other (3)	47/34	NS	NS
12	Karatepe et al. (19)	2009	Turkey, Istanbul	24/5100 (0.47%)	EV (12), Tenia spp. (2), AL (4), Schistosoma Spp. (6)	14/10	18/24 (75%)	36.5
13	Sodergren et al. (20)	2009	United Kingdom	18/1150 (1.57%)	EV (18)	12/6	2/18 (11%)	8-37
14	Ariyarathenam et al. (21)	2010	United Kingdom	13/498 (2.61%)	EV (13)	NS	8/13 (62%)	15
15	Engin et al. (22)	2010	Turkey, Izmir	9/1969 (0.46%)	EV (7), Tenia spp. (2)	8/1	NS	26.4
16	Chandrasegaram et al. (23)	2012	Australia	44/4670 (0.9%)	Not Specified (44)	25/19	18/44 (41%)	NS
17	Gialamas et al. (24)	2012	Greece	7/1085 (0.65%)	EV (7)	4/3	1/7 (17%)	25
18	Hedya et al. (25)	2012	Egypt	11/251 (4.38%)	EV (4), AL (2), Schistosoma Spp. (3), E. Histolytica (2)	3/8	6/11 (55%)	16
19	Zakaria et al. (26)*	2012	Oman	88/1600 (5.5%)	EV (45), Tenia spp. (5), AL (23), Schistosoma Spp. (8), TT (7)	NS	54/88 (61%)	NS
20	Ilhan et al. (27)	2013	Turkey, Izmir	19/3863 (0.49%)	EV (16), Tenia spp. (3)	12/7	9/19 (47%)	30.6
21	Charfi et al. (28)	2014	Tunisia	1599/24697 (6.47%)	EV (1599)	NS	693/1599 (43%)	NS
22	Yabanoglu et al. (29)	2014	Turkey, Ankara	17/1452 (1.17%)	EV (15), E. Histolytica (2)	11/6	4/17 (24%)	36.6 ± 20.1
23	Fleming et al. (30)*	2015	United Kingdom	13/182 (7.14%)	EV (13)	3/10	4/13 (31%)	11
24	Lala and Upadhyay (31)*	2015	New Zealand	109/2923 (4%)	EV (109)	82/27	25/109 (23%)	11
25	Ahmed et al. (32)	2015	Pakistan	85/2956 (2.88%)	EV (84), AL (1)	62/22	24/85 (28%)	24.6
26	Zaghlool et al. (33)	2015	Egypt	6/1536 (0.39%)	EV (4), Schistosoma Spp. (2)	3/3	4/6 (67%)	17
28	Akkapulu et al. (34)	2016	Turkey, Mus	9/1446 (0.62%)	EV (9)	7/2	1/9 (11%)	27 ± 2.9
29	Hamdona et al. (35)	2016	Palestine	30/200 (15%)	EV (30)	17/13	27/30 (90%)	NS
30	Pisoh-Tangnyin et al. (36)	2016	Cameroon	13/112 (11.6%)	EV (5), AL (8)	NS	NS	NS
31	Altun et al. (37)	2017	Turkey, Balikesir	12/660 (1.82%)	EV (9), Tenia spp. (3)	7/5	5/12 (42%)	15
32	Mardani et al. (38)	2017	Iran	31/13744 (0.22%)	EV (31)	21/10	3/31 (10%)	12.5
33	Arham et al. (39)	2018	Pakistan	15/471 (3.18%)	EV (15)	11/4	2/15 (13%)	9.07 ± 9.04
34	Bayoumy et al. (40)	2018	Egypt	6/126 (4.76%)	EV (4), Schistosoma Spp. (2)	NS	4/6 (67%)	NS
35	Zouari et al. (41)*	2018	Tunisia	53/540 (9.81%)	EV (53)	23/30	23/53 (43%)	9.28 ± 2.77
36	Fadiel et al. (42)	2019	Libya	120/175 (68.57%)	EV (8), G. Lamblia (6), E. Histolytica (61), Other (87)	60/60	NS	NS
37	Pehlivanoglu et al. (43)	2019	Turkey, Adiyaman	24/3222 (0.74%)	EV (24)	12/12	8/24 (33%)	12 ± 9.34
38	Tayfur and Balci (44)	2019	Turkey, Erzincan	32/2400 (1.33%)	EV (22), Tenia spp. (10)	23/9	32/32 (100%)	23.68
39	Hasan et al. (45)	2020	Egypt	31/1150 (2.7%)	EV (31)	18/13	1/31 (3%)	NS
40	Sarici et al. (46)	2020	Turkey, Malatya	42/2754 (1.53%)	EV (38), Tenia spp. (2), AL (1), Other (1)	25/17	22/42 (52%)	NS
41	Al-Balas et al. (47)	2021	Jordan	14/1510 (0.93%)	EV (12), E. Histolytica (2)	9/5	3/14 (21%)	11.4
42	Gumus and Sogutcu (48)	2021	Turkey, Diyarbakir	268/14797 (1.8%)	EV (268)	135/133	85/268 (32%)	NS
43	Kosmaz et al. (49)	2021	Turkey, Ankara	24/7344 (0.33%)	EV (22), A. Lumbricoides (2)	12/12	8/24 (33%)	33
44	Sousa et al. (50)	2021	United States	38/3541 (1.07%)	EV (38)	20/18	30/38 (78.3%)	NS

**EV:** Enterobius vermicularis, **AL:** Ascaris lumbricoides, **TT:** Trichuris trichiura, **B. Coli:** Balantidium coli, **E. Histolytica:** Entamoeba histolytica **NS:** Not Specified, * paediatric research

**Table 2:** Literature review summary: Seeds in the appendix

**No**	**Author**	**Publication Year**	**Seed Type, Case; n**	**Age - Gender**
1	Prescott O. (51)	1816	Cocoa or Chocolate Nut; 1	42-M
2	Jacobi A. (52)	1887	Berry Seed; 1	Not specified
3	Hupp FL. (53)	1899	Grape Seed; 1 or 2	Not specified
4	Mitchell LJ. (54)	1904	Grape Seed; 8	43-M, 42-M, 33-M, 29-F, 26-M, 52-F, 35-M, 23-M
5	Barnett and Macfie (55)	1907	Clove; 1	58-M
6	Bidwell LA. (56)	1911	Grape Seed; 2, Fig Seed; 1, Rose Tree Seed;1	Not specified
7	Wright T. (57)	1914	Grape Seed, 1	Not specified
8	Balch CM. (58)	1971	Not Specified; 12, Barcelona Nut; 2, Oat; 2, Grape Seed; 2, Pinon Nut Seed;1, Barley;1, Caraway;1, Date;1, Raisin;1, Fig;1	Not specified
9	Byard et al. (59)	1998	Not Specified; 1	3-F
10	Koseogullari et al. (60)	2006	Watermelon; 1	9-M
11	Hulme P. (61)	2010	Not Specified; 1	13-M
12	Engin et. al. (62)	2011	Not Specified fruit seed;1 undigested plant residual; 7	Not specified
13	Campora et al. (6)	2017	Not Specified; 5	Not specified
14	Grillo et al. (7)	2021	Not Specified; 13	Not specified

This study was approved by the Local Ethics Committee (decision no: 07/15, decision date: 24.05.2021) and conducted in accordance with the principles set forth in the Declaration of Helsinki.

### Statistical Analysis

Data obtained as a result of the study were analyzed statistically. Continuous variables were expressed as mean ± standard deviation (SD) values, and categorical variables were expressed as numbers (n) or percentage (%) values where appropriate.

## RESULTS

The H&E slides of 9,480 appendectomy specimens were re-evaluated. Adult or ova of parasites were observed in 56 (0.6%) cases, of whom 37 were female and 19 were male. The male-to-female ratio was 1:1.9, and the mean age was 23 years (2-56 years). EV was seen in 45, and *Taenia spp.* were seen in 11 cases. The mean and median ages of the patients with EV and *Taenia spp.* were 19-14 years and 40-45 years, respectively. All patients with parasitic infestation presented with right lower quadrant pain. The ultrasonographic findings were compatible with acute appendicitis in 41 patients and suspected acute appendicitis in 15 patients. The mean diameter of the appendectomy specimens was 0.7 cm (range, 0.4-1 cm). In 32 cases, the appendix lumen was filled with feces and enlarged. Active inflammation was observed in 16 cases. Three of these cases also had perforation. In 40 cases, there was no histological evidence of appendicitis. The demographic data and histopathological features of the cases are summarized in Table 3.

**Table 3:** Detailed Characteristic of the cases with parasites, n

	**This study** **Patients, n**	**Gender** **(F/M)**	**Mean Age (Range)**	**Additional Findings**	
				Negative appendectomy*, n	**Acute Appendicitis, n**
*Enterobius vermicularis*	45	27/18	18.7 years (2-56)	32	13
*Tenia spp.*	11	10/1	40 years (17-53)	8	3
Total	56	37/19	22.9 years	40	16

*Negative appendectomy: appendectomy specimen without any inflammatory change

**Figure 1:**
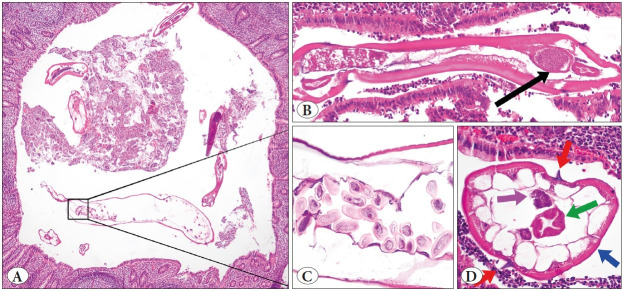
Cross-section of Enterobius vermicularis (EV) in the appendix lumen (**A;** H&E stain, ×40), adult EV with a thick cuticle, lateral alae, and visible organs (**B;** composite photograph of H&E stain ×100, gastrointestinal organs; black arrow); longitudinal section of the uterine reproductive system of female EV; it is filled with multiple D-shaped ova (**C;** H&E stain, x400) and adult EV at higher magnification (**D;** bilateral spikes (alae); red arrows, the cuticle; blue arrow, reproductive organ; pink arrow, gastrointestinal organ; green

**Figure 2:**
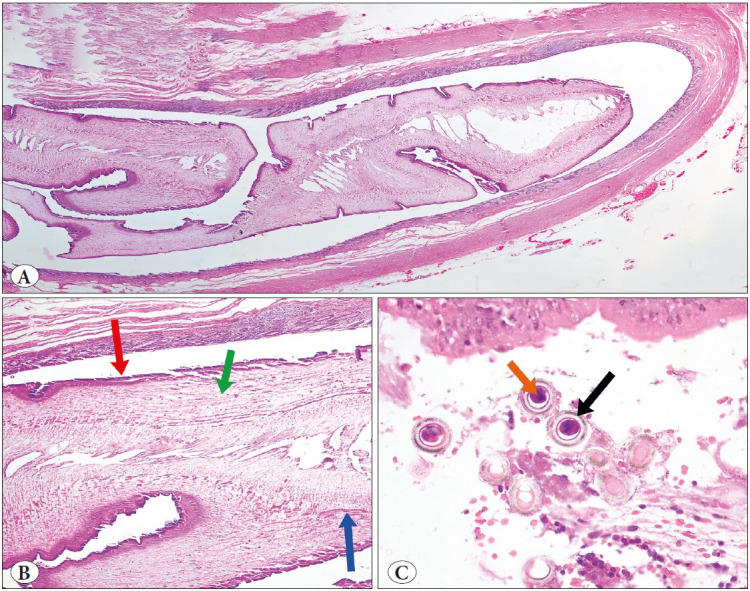
Cross-section of the adult Taenia spp. in the appendix lumen (**A;** composite photograph of H&E, ×100), a section of the adult Taenia spp. at higher magnification (**B;** thick outer tegument with microvillus; red arrow, calcareous corpuscles; green arrow, smooth muscle; blue arrow H&E stain, ×100), and Taenia ova in in the appendix lumen; spherical brown structures and a large round eosinophilic center (orange arrow) encircled by brown rings (black arrow) (**C;** H&E stain, ×400)

In cases with EV, 1 to 10 adult forms were seen in the appendix lumens, which were 2 mm to 6 mm x 0.2 mm to 0.4 mm in size, and in which cuticle structures, gastrointestinal or reproductive organs, and lateral ales could be detected on the outer parts. Additionally, a D-shaped egg form was observed in the reproductive system of the female forms. In three of the cases with Taenia, adult forms with gravid proglottid were observed in the appendix lumen, whereas in the other eight cases only eggs form were observed. Images of some cases with EV and Taenia are given in Figure 1 and Figure 2.

Plant seeds were detected in 47 appendectomy specimens. Of the 47 cases with seeds, 21 were female, and 26 were male.

**Figure 3:**
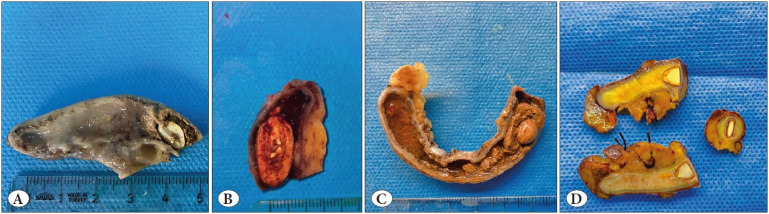
Gross appearance of appendectomy specimens: Lemon seed surrounded by fecaloid in the proximal part of appendix lumen **(A)**, Non specified seed fragments in fecaloid **(B)**, appendix lumen obstructed by cherry pip surrounded with fecaloid **(C)**, Lemon seed in the proximal portion of the appendix **(D)**

The mean age of these cases was 26 years (median; 24, range; 15-53 years). Seeds were detected during macroscopic examination in five cases: olive seed in one case, lemon seed in two cases, and cherry seed in the remaining two cases (Figure 3). In the remaining 42 cases, the seeds were detected during the histopathological examination. Active inflammation was present in 35 cases, whereas no evidence of inflammation was observed in the other 12 cases.

**Figure 4:**
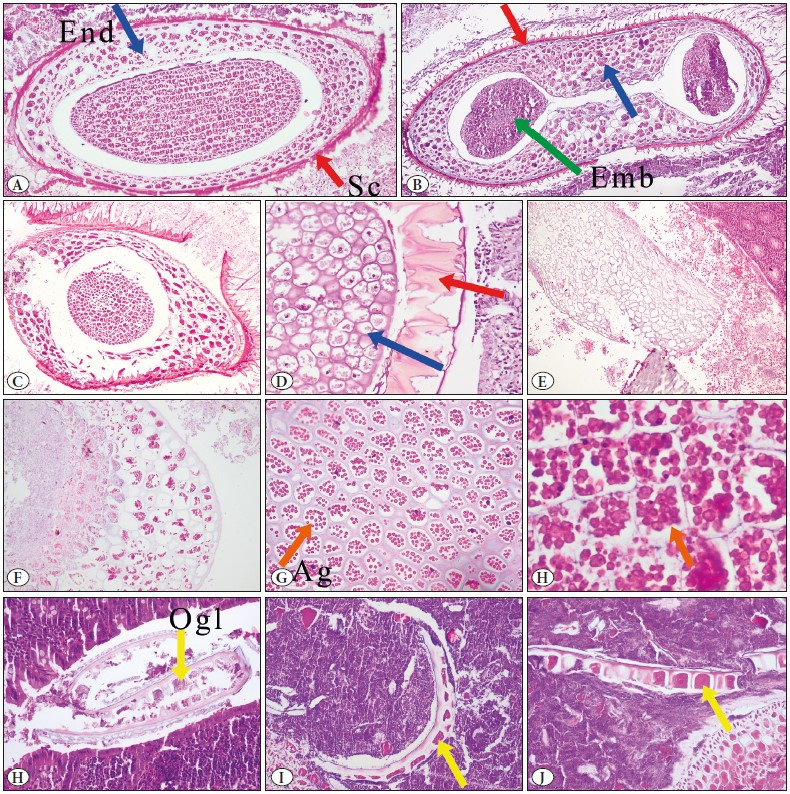
Cross-section of seeds in in the appendix lumen; the seed coat (Sc); red arrow, endosperm (End), and embryo (Emb); green arrow (**A, B and C;** H&E stain, x100), partly digested seed (**D;** H&E stain, x200), digested plant-seed fragment with empty endosperm region (**E;** x40), partly digested plant seed, endosperm filled with starch and fat (**F;** H&E stain, x100) and the endosperm region containing various-sized bright pink-red colored spherules (Ag:aleurone grains); orange arrow (**G;** H&E stain, x200 and H, H&E stain, x400). Convoluted, Small grain fragments; thick, the outer grain layer (Ogl: the outermost layer of the endosperm); yellow arrow (**H, I, and J;** H&E stain, x400).

The seed-containing cases were evaluated under the guidance of a botanist. Consequentially, it was thought that the seeds in these cases might pertain to grasses such as tomato, pepper, kiwi, apple, lentil, and oat. It could not be possible to determine the origin of the seeds in all cases. Examples of basic anatomical structures of the seeds, i.e., seed coat located in the outer part of the nucleus, endosperm region that surrounds the embryo and provides nutrition, embryo structures, and convoluted cereal parts similar to parasites, are presented in Figure 4.

**Figure 5:**
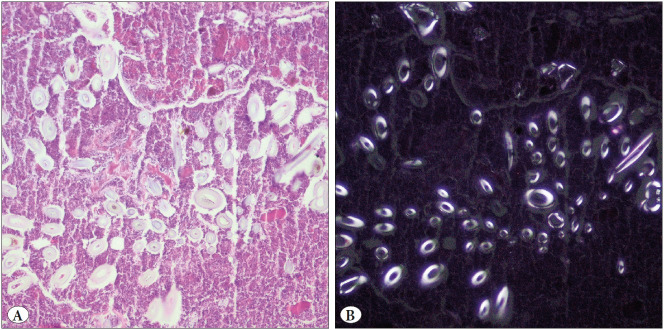
Transparent tube-like foreign body structure in fecaloid (**A;** H&E stain, x100), under polarized light **(B)**

Foreign bodies were detected in two cases. There was no history of swallowing an object in these two cases. Both patients, 25 and 32 years old, presented with right lower abdominal pain lasting for 1-2 days. The white blood cell count was 10x109/L and 9.5x109/L, respectively. In the USG (ultrasonography) examination, the diameters of the appendix lumens were increased; thus, they were interpreted as acute appendicitis. No finding indicated a foreign body in the abdominal X-ray examinations. A plastic T-shaped structure was observed within the fecalith during the macroscopic examination in one of the cases. In contrast, transparent white small tube-like foreign body structures were observed during histopathological examination in the other case (Figure 5).

## DISCUSSION

**Figure 6:**
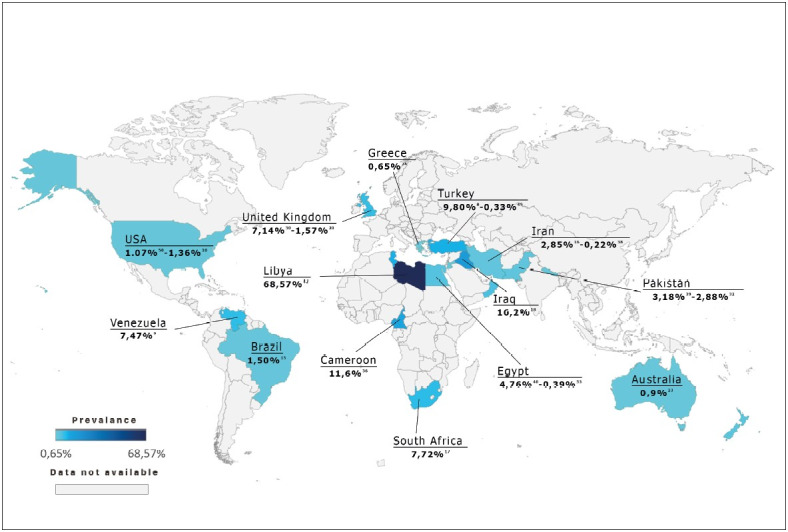
World map; presenting prevalence of parasites in appendectomy specimens by countries. * *This map was created in Excel format with the data of the studies in Table-1. Its color is based on the study in which the highest prevalence of that country was reported. The data and the reference numbers of the studies were added to the map with photoshop.*

Parasites, seeds, and foreign bodies are rarely seen in appendectomy specimens. The results of the literature review (Table 1 and Figure 6) revealed that the prevalence of parasites in appendectomy specimens varied immensely between studies (8–50). The highest prevalence was reported in a study from Libya and the lowest prevalence was reported from Iran, with 68.6% and 0.22%, respectively (38,42). The prevalence ranged between 0.33% and 9.8% in studies from Turkey (8,49). In our study, parasites were observed in 0.6% (56/9.480) of the appendectomy specimens. Some studies suggested that prevalence could be associated with the country’s income level, developmental level, and hygiene conditions (18,24,63). However, since the prevalence is affected by many factors (the experience of the researcher, number of pieces taken during macroscopic sampling, the age range of the population, etc.), we believe that well-designed prospective studies are required to address the association between them.

The most common parasitic agent in the appendix has been reported as EV, while Tenia was the second most common in several studies, as in our series (15,19,22,26,37). Other parasitic agents such as *Entamoeba histolytica*, *Balantidium coli*, *Entamoeba histolytica*, *Schistosoma*, and *Ascaris lumbricoides* have been indicated in a few studies (2), though none of these were observed in the current study.

In our study, there was a significant difference in patient age between EV and *Tenia spp.*; the mean and median ages were 19-14 years for EV and 40-45 years for *Tenia spp.* EV infection tended to occur at a younger age than *Tenia spp.*, consistent with previous studies (16,44).

Patients with parasite often presented with appendicitis-like symptoms such as right lower quadrant pain, vomiting, and loss of appetite in our study. However, appendicitis findings were observed in only 28% of cases. Furthermore, there was no significant correlation between the presence of appendicitis and the parasitic agent type. The literature review showed that the rates of inflammation accompanying parasitic infestation reported in different studies varied greatly, between 3% and 100% (44,45). The fact that inflammation is not observed in every case raises to question of whether the presence of parasites in the appendix is coincidental or a factor that triggers inflammation. It has been reported that the parasites that involve the mucosa or invade the lamina propria may trigger inflammation (1,44). However, the relationship between parasites and ova found in the appendiceal lumen and the development of appendicitis-like symptoms has not yet been fully elucidated. As with foreign bodies, parasites in the appendiceal lumen can induce fecal concretion. Grimes et al. suggested that the presence of fecaliths could lead to abdominal pain without inflammation (64). In the light of this information, appendicitis-like symptoms in the cases with parasites may be related to the increased feces concentration and the development of fecaliths.

Foreign bodies are also rarely encountered in appendectomy specimens (4). Most undigested foreign bodies pass through the gastrointestinal system and are excreted without any complication. However, materials with sharp and thin ends may cause perforation of the appendix. On the other hand, blunt-ended foreign bodies may not pass into the colon after entering the appendix (4). Various materials including retained shotgun pellets, teeth, mercury, eggshells, and gallstones have been detected in the appendix (4). Most of these materials are radiopaque; thus, they can be detected in preoperative radiographic evaluation (4). As is the case with foreign bodies, large seeds, such as those of olives, cherries, and lemons pips, cannot be redirected to the colon after they enter the appendiceal lumen and may thus cause appendicitis by obstructing the appendiceal lumen. Tiny seeds are usually determined incidentally during histopathological examination.

The literature review results have indicated that most studies on this subject were carried out in the early nineteenth century (51–57). In those years, it was thought that seeds played an important role in developing appendicitis (65). In the following years, relatively few studies addressed the seeds, which may be attributed to the fact that the observation of seeds in the appendix lumen did not change the treatment plan or follow-up approaches, and thus that the studies aimed at such findings did not receive enough attention from researchers for publication purposes. To our best knowledge, only 63 cases have been described in the literature to date (6,7,51–62). The details of these cases are summarized in Table 2. The highest incidence was reported in the study of Grillo et al., in which complete and fragmented seed parts were found in 13 (2.2%) of the 588 appendicectomy specimens (7). In this study, we determined seeds in 47 (0.5%) of the cases. It was thought that the seeds might pertain to plants such as tomato, pepper, kiwi, apple, lentil, and oat, in addition to olive, lemon, and cherry. The mean age of the patients was 26 years and the seeds were frequently observed in young adults (median age; 24 years). Only 3 patients were younger than 16 years.

Fruits, vegetables, and undigested food particles can be seen in the histological or cytological examination of surgical pathology specimens, and some have been documented in the literature as potential mimickers of clinically significant findings (66,67). In the gastrointestinal tract, seeds that can adhere to the intestinal mucosa with the glycoprotein-rich villi available on their surfaces may be mistakenly identified as parasites due to their complex and unfamiliar histological appearance, leading to a misdiagnosis (68). Knowledge of the morphological features of such substances is crucial to distinguish them from parasites and drug residues which otherwise would require additional treatment. Routine pathology practices include differential analysis of the food residues from the structures they can mimic. However, specific seed type identification may be necessary for forensic pathology practices, such as determining the victim’s last meal (69).

Seeds are multifaceted so that they can exhibit wide variations under the microscope. In this context, seed fragments may lead to the suspicion of the presence of helminths, such as *Anisakis simplex* (5). Non-expert pathologists may misidentify seed structures as unusual parasitic agents. Grillo et al. reported that three cases whose specimens included seeds were referred by the pathologists who considered that the seeds could be some un-identified/unrecognized worm (7). We also observed that some seed photographs were mistakenly published as parasites in the literature (35,39,70).

The mature seed comprises three parts: seed coat, embryo, and endosperm structures (5,6). However, the thickness, the color, and the shape of these structures may differ between species (7). The outer covering of a seed is called the seed coat. Seed coats help protect the embryo from external factors. The endosperm contains bright pink-red colored starch and fat globules in various sizes that surround and nourish the embryo with one or two cotyledons (6,7). Fragmented-semi-digested seed structure, particularly curly particles of cereals, may raise suspicion of parasites (5). Parasites are usually smaller in size than seeds. Females of EVs are often 8-13 mm long, and the males 2-5 mm long. There is a thick cuticle on the outside and “lateral alae” that protrude like spines on the surface. Gastrointestinal and genitourinary organs could be observed. In the reproductive organs of females, 50-60 µm by 20-30 µm “D-shaped” eggs can be observed (71). *Taenia spp.*, generally 2-12 mm in length, are rarely observed in appendectomy specimens (2,3). In most cases, the egg form is seen in the lumen. Taenia egg, which has a spherical shape, is 30-40 µm in diameter. Depending on the level of the slice, the egg may appear in different specimens as spherical brown structures or large round eosinophilic centers encircled by brown rings. Adult worms of Taenia have many proglottids (1000 to 2000). Mature proglottids each have genital organs consisting of about 300 to 400 testes and a vaginal sphincter (71).

## CONCLUSION

Parasites, foreign bodies, and plant seeds are rarely found in the appendix, and if present, they are usually detected incidentally during the histopathological examinations of the appendectomy specimens. Fragmented seeds can exhibit wide variations under the microscope, and their histopathological images can mimic parasites. In such cases, assessing the above-mentioned histopathological features will be beneficial for differential diagnosis between parasites and seeds.

## Conflict of Interest

The author(s) declared no potential conflicts of interest with respect to the research, authorship, and/or publication of this article.

## Funding

The author(s) received no financial support for this article's research, authorship, and/or publication.

## Figures and Tables

**Appendix 1:**
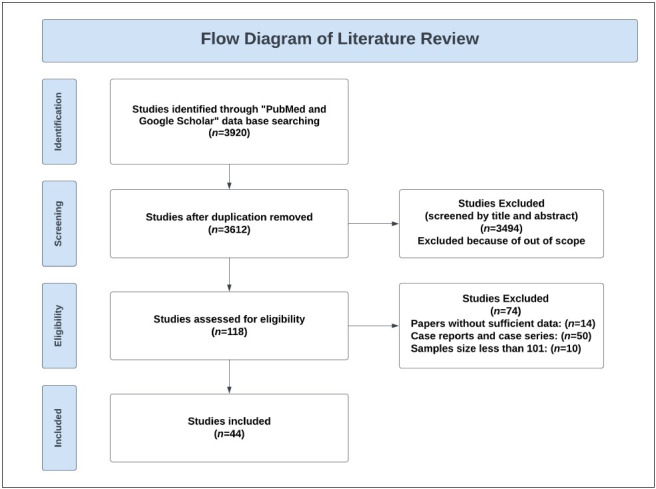
Flow diagram of literature review

## References

[ref-1] Harp James A. (2003). Parasitic infections of the gastrointestinal tract. Curr Opin Gastroenterol.

[ref-2] Eslahi Aida Vafae, Olfatifar Meysam, Houshmand Elham, Abdoli Amir, Bijani Behzad, Hashemipour Sima, Mahmoudi Razzagh, Hajialilo Elham, Javad Abbaszadeh Afshar Mohammad, Mohammadzadeh Ali Reza, Badri Milad (2022). Parasites in surgically removed appendices as a neglected public health concern: a systematic review and meta-analysis. Pathog Glob Health.

[ref-3] Issin Gizem, Demir Fatih, Simsek Hasan Aktug, Cagatay Diren Vuslat, Tayfur Mahir, Balci Mecdi Gurhan (2023). Retrospective analysis of the appendiceal neoplasms: sampling technique may influence neoplasm detection. Postgrad Med J.

[ref-4] Klingler P. J., Seelig M. H., DeVault K. R., Wetscher G. J., Floch N. R., Branton S. A., Hinder R. A. (1998). Ingested foreign bodies within the appendix: A 100-year review of the literature. Dig Dis.

[ref-5] Razzano Dana, Gonzalez Raul S. (2020). Disease, drugs, or dinner? Food histology can mimic drugs and parasites in the gastrointestinal tract. Virchows Arch.

[ref-6] Campora Michela, Trambaiolo Antonelli Chiara, Grillo Federica, Bragoni Alberto, Cornara Laura, Migliora Paola, Pigozzi Simona, Mastracci Luca (2017). Seeds in the appendix: a 'fruitful' exploration. Histopathology.

[ref-7] Grillo Federica, Campora Michela, Cornara Laura, Cascini Alberta, Pigozzi Simona, Migliora Paola, Sarocchi Francesca, Mastracci Luca (2021). The Seeds of Doubt: Finding Seeds in Intriguing Places. Front Med (Lausanne).

[ref-8] Eğilmez R, Saygı G, Aker H, Elagöz Ş (2000). Retrospective analysis of appendix vermiformis specimens for intestinal helminths. Türkiye Ekopatoloji Derg.

[ref-9] Dorfman Saul, Cardozo José, Dorfman Denny, Del Villar Alonso (2003). The role of parasites in acute appendicitis of pediatric patients. Invest Clin.

[ref-10] Arca Marjorie J., Gates Robert L., Groner Jonathan I., Hammond Sue, Caniano Donna A. (2004). Clinical manifestations of appendiceal pinworms in children: an institutional experience and a review of the literature. Pediatr Surg Int.

[ref-11] Yildirim Sedat, Nursal Tarik Z., Tarim Akin, Kayaselcuk Fazilet, Noyan Turgut (2005). A rare cause of acute appendicitis: parasitic infection. Scand J Infect Dis.

[ref-12] Fallah E, Dehgani A (2011). A Study on entrobius vermicularis infection in a appendices removed by surgery in Tabriz Hospitals. Internet J Parasit Dis.

[ref-13] Sah Shatrughan Prasad, Bhadani Punam Prasad (2006). Enterobius vermicularis causing symptoms of appendicitis in Nepal. Trop Doct.

[ref-14] Aydin Ozgür (2007). Incidental parasitic infestations in surgically removed appendices: a retrospective analysis. Diagn Pathol.

[ref-15] Silva Danielle Fernandes, Silva Reinaldo José, Silva Márcia Guimarães, Sartorelli Alesso Cervantes, Rodrigues Maria Aparecida Marchesan (2007). Parasitic infection of the appendix as a cause of acute appendicitis. Parasitol Res.

[ref-16] Ramezani Mohammad Arash, Dehghani Mahmoud Reza (2007). Relationship between Enterobius vermicularis and the incidence of acute appendicitis. Southeast Asian J Trop Med Public Health.

[ref-17] Chamisa I. (2009). A clinicopathological review of 324 appendices removed for acute appendicitis in Durban, South Africa: a retrospective analysis. Ann R Coll Surg Engl.

[ref-18] Al-Shadood HAS, Sultan BA, Alsaiegh AM (2009). Parasitic cause of acute appendicitis in Najaf. Kufa Medical Journal.

[ref-19] Karatepe O., Adas G., Tukenmez M., Battal M., Altiok M., Karahan S. (2009). Parasitic infestation as cause of acute appendicitis. G Chir.

[ref-20] Sodergren Mikael H., Jethwa Paras, Wilkinson Simon, Kerwat Rajab (2009). Presenting features of Enterobius vermicularis in the vermiform appendix. Scand J Gastroenterol.

[ref-21] Ariyarathenam A. V., Nachimuthu S., Tang T. Y., Courtney E. D., Harris S. A., Harris A. M. (2010). Enterobius vermicularis infestation of the appendix and management at the time of laparoscopic appendectomy: case series and literature review. Int J Surg.

[ref-22] Engin O., Calik S., Calik B., Yildirim M., Coskun G. (2010). Parasitic appendicitis from past to present in Turkey. Iran J Parasitol.

[ref-23] Chandrasegaram Manju D., Rothwell Lincoln A., An Ethan I., Miller Rose J. (2012). Pathologies of the appendix: a 10-year review of 4670 appendicectomy specimens. ANZ J Surg.

[ref-24] Gialamas Eleftherios, Papavramidis Theodossis, Michalopoulos Nick, Karayannopoulou Georgia, Cheva Angeliki, Vasilaki Olga, Kesisoglou Isaak, Papavramidis Spiros (2012). Enterobius vermicularis: a rare cause of appendicitis. Turkiye Parazitol Derg.

[ref-25] Hedya Mohamed Saied, Nasr Magid Mahmoud, Ezzat Hussein, Hamdy Hussam Mohamed, Hassan Ahmed Mohamed Abdelaziz, Hammam Olfat (2012). Histopathological findings in appendectomy specimens: a retrospective clinicopathological analysis. J Egypt Soc Parasitol.

[ref-26] Zakaria Ossama M., Zakaria Hazem M., Daoud Mohamed Yasser, Al Wadaani Hamed, Al Buali Waleed, Al-Mohammed Hamdan, Al Mulhim Abdulrahman S., Zaki Wafaa (2013). Parasitic infestation in pediatric and adolescent appendicitis: a local experience. Oman Med J.

[ref-27] Ilhan Enver, Senlikci Abdullah, Kızanoglu Hale, Ustüner Mehmet Akif, Vardar Enver, Aykas Ahmet, Yeldan Eyup, Yıldırım Mehmet (2013). Do intestinal parasitic infestations in patients with clinically acute appendicitis increase the rate of negative laparotomy? Analysis of 3863 cases from Turkey. Prz Gastroenterol.

[ref-28] Charfi Slim, Sellami Ahmad, Affes Abdellatif, Yaïch Khalil, Mzali Rafik, Boudawara Tahya Sellami (2014). Histopathological findings in appendectomy specimens: a study of 24,697 cases. Int J Colorectal Dis.

[ref-29] Yabanoğlu Hakan, Aytaç Hüseyin Özgür, Türk Emin, Karagülle Erdal, Calışkan Kenan, Belli Sedat, Kayaselçuk Fazilet, Tarım Mehmet Akın (2014). Parasitic infections of the appendix as a cause of appendectomy in adult patients. Turkiye Parazitol Derg.

[ref-30] Fleming C. A., Kearney D. E., Moriarty P., Redmond H. P., Andrews E. J. (2015). An evaluation of the relationship between Enterobius vermicularis infestation and acute appendicitis in a paediatric population--A retrospective cohort study. Int J Surg.

[ref-31] Lala Shareena, Upadhyay Vipul (2016). Enterobius vermicularis and its role in paediatric appendicitis: protection or predisposition?. ANZ J Surg.

[ref-32] Ahmed Muhammad Umer, Bilal Muhammad, Anis Khurram, Khan Ali Mahmood, Fatima Kaneez, Ahmed Iqbal, Khatri Ali Mohammad, Shafiq-ur-Rehman null (2015). The Frequency of Enterobius Vermicularis Infections in Patients Diagnosed With Acute Appendicitis in Pakistan. Glob J Health Sci.

[ref-33] Zaghlool Dina A., Hassan Amal A., Ahmed Mona A., Faidah Hani S. (2015). Incidental parasitic infections in surgically removed appendices: A retrospective analysis. J Egypt Soc Parasitol.

[ref-34] Akkapulu N., Abdullazade S. (2016). Is Enterobius vermicularis infestation associated with acute appendicitis?. Eur J Trauma Emerg Surg.

[ref-35] Hamdona Shereen M., Lubbad Abdel Monem, Al-Hindi Adnan I. (2016). Histopathological study of Enterobius vermicularis among appendicitis patients in Gaza strip, Palestine. J Parasit Dis.

[ref-36] Pisoh-Tangnyin C, Kamga Fhl, Kechia Fa, Laah Ns, Guifo Ml, Takongmo S (2016). Intestinal helminths in some cases of acute appendicitis operated in Bamenda, Cameroon. African J Clin Exp Microbiol.

[ref-37] Altun Eren, Avci Veli, Azatcam Meltem (2017). Parasitic infestation in appendicitis. A retrospective analysis of 660 patients and brief literature review. Saudi Med J.

[ref-38] Mardani Ahmad, Feizi Fatemeh, Fakhar Mahdi, Beyranvand Hossein Barani, Farrokhi Mohsen, Abbasi Mohammad, Asfaram Shabnam (2017). Enterobius vermicularis infection among appendectomy specimens in Qom Province, Central Iran: A retrospective study. Comp Clin Pathol.

[ref-39] Arham M, Arish M, Khan JS (2018). Incidental parasitic infestations in surgically removed appendices and its association with inflammation. J Rawalpindi Med Coll.

[ref-40] Bayoumy Ahmed M., Elnasr Adel O. Seif, Ibrahim Ibrahim A. (2018). Role and incidence of parasitic infection in adult Egyptian patients with acute appendicitis. Egypt J Hosp Med.

[ref-41] Zouari Mohamed, Louati Hamid, Abid Imen, Trabelsi Fatma, Ben Dhaou Mahdi, Jallouli Mohamed, Mhiri Riadh (2018). Enterobius vermicularis: A Cause of Abdominal Pain Mimicking Acute Appendicitis in Children. A Retrospective Cohort Study. Arch Iran Med.

[ref-42] Fadiel MM, Elsamad NAA, Younis EZ (2019). Parasitic infection in surgically removed appendices from patients in Al-Jalal Hospital Benghazi, Libya. Biomed J Sci Tech Res.

[ref-43] Pehlivanoğlu Burçin, Aydın Türk Bilge, İşler Serap, Özdaş Sabri, Abeş Musa (2019). Findings in Appendectomies with Enterobius vermicularis Infection: Pinworm Is Not A Cause of Appendicitis. Turkiye Parazitol Derg.

[ref-44] Tayfur M., Balci M. G. (2019). Pathological changes in appendectomy specimens including the role of parasites: A retrospective study of 2400 cases of acute appendicitis. Niger J Clin Pract.

[ref-45] Hasan Abdulkarim, Nafie Khalid, El-Sayed Samar, Nasr Mohamed, Abdulmohaymen Ayman, Baheeg Mohamed, Abbadi Osama (2020). Enterobius vermicularis in appendectomy specimens; Clinicopathological assessment: Cross sectional study. Ann Med Surg (Lond).

[ref-46] Sarici Baris, Akbulut Sami, Ozcan Mehmet, Demyati Khaled, Samdanci Emine (2020). Unusual infectious agents detected in appendectomy specimens: A retrospective analysis of 42 cases. Turk J Surg.

[ref-47] Al-Balas Hamzeh, Al-Saffar Raith S., Al-Balas Mahmoud, Al-Wiswasy Mohammad K. M., Abu Salhiyeh Ala'a, Al-Sharqi Yasmeen, Yousuf Mustafa Saad, Bani-Hani Kamal (2021). Unusual histopathological findings in appendectomy specimens with clinical diagnosis of acute appendicitis: A retrospective cohort analysis. Ann Med Surg (Lond).

[ref-48] Gümüş Serdar, Söğütçü Nilgün (2021). Parasitic Appendicitis in 14.797 Cases: A Retrospective Cohort Study. Turkiye Parazitol Derg.

[ref-49] Koşmaz Koray, Şenlikci Abdullah, Süleyman Marlen, Durhan Abdullah (2021). Parasitic infestation in patients undergoing appendectomy: Retrospective analysis of 7,344 cases. Turkish J Color Dis.

[ref-50] Sousa John, Hawkins Russell, Shenoy Archana, Petroze Robin, Mustafa Moiz, Taylor Janice, Larson Shawn, Islam Saleem (2022). Enterobius vermicularis-associated appendicitis: A 22-year case series and comprehensive review of the literature. J Pediatr Surg.

[ref-51] Prescott Oliver (1816). Case of Fatal Colic from the Lodgement of a Chocolate Nut in the Appendicula Vermiformis. Lond Med Phys J.

[ref-52] Jacobi A (1890). The intestinal diseases of infancy and childhood: Physiology, hygiene, pathology and therapeutics.

[ref-53] Hupp FL (1899). The present status of appendicitis: With report of forty-five cases.

[ref-54] Mitchell LJ (1904). A series of foreign bodies in the vermiform appendix met with in 1,600 necropsies.

[ref-55] Barnett W.Hal, Macfie J.W.Scott (1907). A case of Appendicitis excited by a clove, the appendix being the sole viscus in a hernial sac. The Lancet.

[ref-56] Bidwell LeonardA. (1911). Foreign bodies in the vermiform appendix. The Lancet.

[ref-57] Wright Thos (1914). The craze for appendicectomy. BMJ.

[ref-58] Balch C. M., Silver D. (1971). Foreign bodies in the appendix. Report of eight cases and review of the literature. Arch Surg.

[ref-59] Byard R. W., Manton N. D., Burnell R. H. (1998). Acute appendicitis in childhood: did mother know best? A pathological analysis of 1409 cases. Med J Aust.

[ref-60] Köseoğulları AA, Özel ŞK, Bakal Ü, Kazez A (2006). Apandisitin nadir bir nedeni: Meyve çekirdeği.

[ref-61] Hulme Peter (2010). Foreign body causing perforation of the appendix in an African boy. Pan Afr Med J.

[ref-62] Engin Omer, Yildirim Mehmet, Yakan Savas, Coskun Gulnihal Ay (2011). Can fruit seeds and undigested plant residuals cause acute appendicitis. Asian Pac J Trop Biomed.

[ref-63] Taghipour Ali, Olfatifar Meysam, Javanmard Ehsan, Norouzi Mojtaba, Mirjalali Hamed, Zali Mohammad Reza (2020). The neglected role of Enterobius vermicularis in appendicitis: A systematic review and meta-analysis. PLoS One.

[ref-64] Grimes Caris, Chin Diana, Bailey Catherine, Gergely Szabolcs, Harris Adrian (2010). Appendiceal faecaliths are associated with right iliac fossa pain. Ann R Coll Surg Engl.

[ref-65] Andrews Edmund (1896). Do grape seeds cause appendicitis?. JAMA.

[ref-66] Chang S, Moatamed NA, Christina KY, Salami N (2013). The sheep in wolf's clothing: Vegetable and fruit particles mimicking cells and microorganisms in cytology specimens. J Cytol Histol.

[ref-67] Nowacki Nicholas B., Arnold Michael A., Frankel Wendy L., Harzman Alan, Limketkai Berkeley N., Yearsley Martha M., Arnold Christina A. (2015). Gastrointestinal tract-derived pulse granulomata: clues to an underrecognized pseudotumor. Am J Surg Pathol.

[ref-68] Campora Michela, Cornara Laura, Paudice Michele, Mastracci Luca, Grillo Federica (2019). Yellow Is the New Black. Int J Surg Pathol.

[ref-69] Aquila Isabella, Ausania Francesco, Di Nunzio Ciro, Serra Arianna, Boca Silvia, Capelli Arnaldo, Magni Paola, Ricci Pietrantonio (2014). The role of forensic botany in crime scene investigation: case report and review of literature. J Forensic Sci.

[ref-70] Zarbaliyev Elbrus, Celik Sebahattin (2018). Parasitic Appendicitis: A Novel Laparoscopic Approach for the Prevention of Peritoneal Contamination. Can J Infect Dis Med Microbiol.

[ref-71] Ash LR, Orihel TC (2007). Atlas of human parasitology.

